# Supplementary figures and images for: Trained recurrent neural networks develop phase-locked limit cycles in a working memory task
Source: PLoS Comput Biol. 2024 Feb 5;20(2):e1011852. doi: 10.1371/journal.pcbi.1011852 (PMC10868787; doi:10.1371/journal.pcbi.1011852)

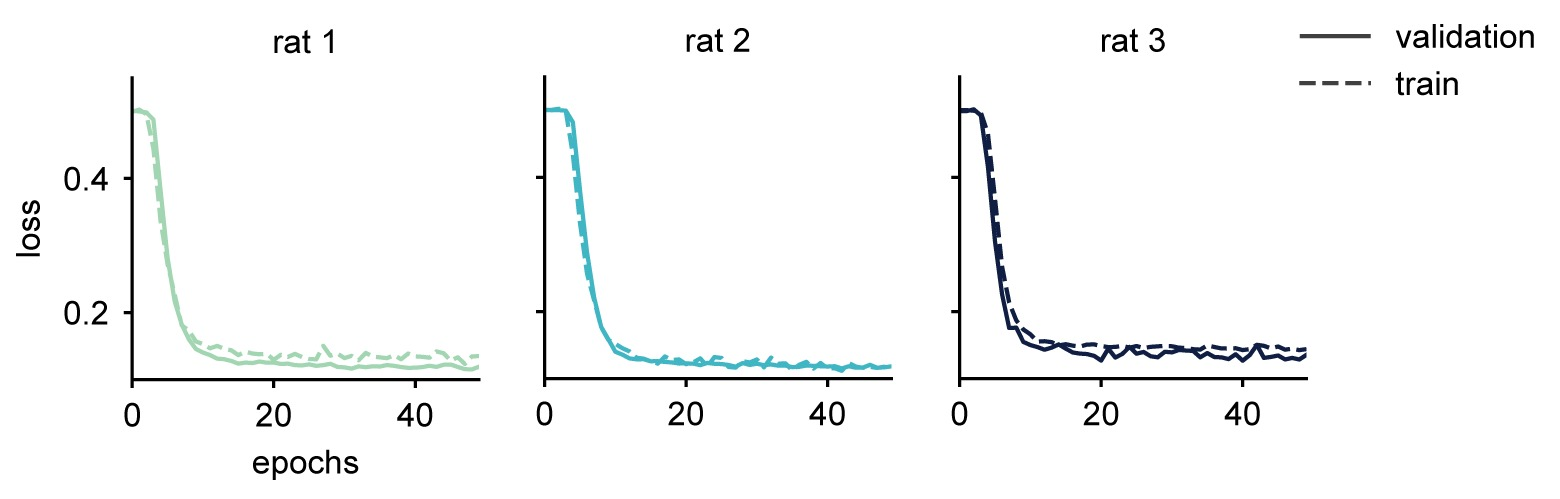

Supplement: S1 Fig — Loss over epochs for three models, each trained with LFP data from a separate rat. An epoch denotes one pass through all trials in the training or validation set. The validation trials are defined before training and are not used for calculating gradients. However, the training and validation error are almost identical throughout training. This is expected, as the validation and training trials only differ in the portion of the local field potential that was used as reference signal for the network, and in the seed used for generating the randomised stimulus onsets and offsets. (TIF) [file pcbi.1011852.s001.tif]

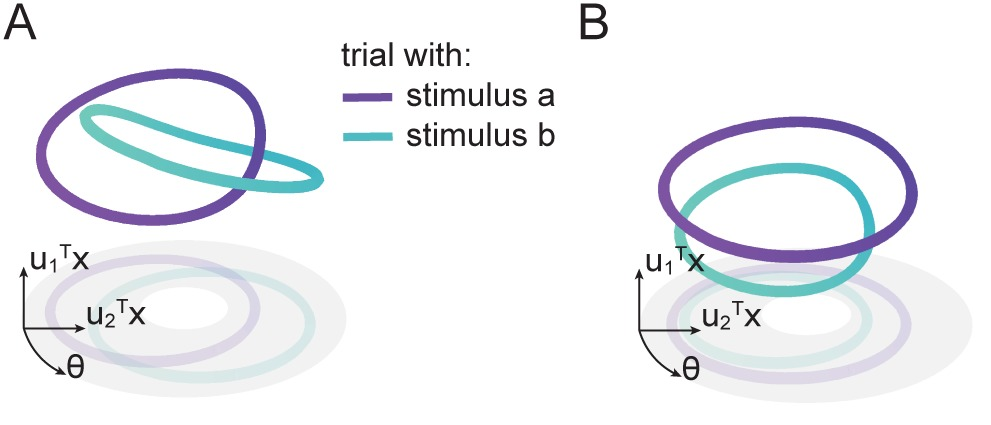

Supplement: S2 Fig — A) We also trained RNNs without rank constraint. For these networks initial entries in the recurrent weight matrix J were drawn from a zero-mean Gaussian with variance g2N. Here, we add a regularisation term to the loss, which keeps the average firing rates close to 0, to avoid a rate-coding solution (Eq 6). We set g to 0.6 and took a learning rate of 0.001, with the training setup otherwise as for the low-rank networks (Training). In order to find a basis similar to the one used for plotting the dynamics of the low-rank RNN we took the following approach. First we calculated basis vectors for the activity due to recurrent dynamics: J tanh(x(t)), by performing a Principal Component Analysis (singular vector decomposition): UΣVT=Jtanh(X), where X is an N×2T matrix containing the activity of all units for one period of oscillation of each type of trial (stimulus a and b). We took the first two columns (principal components), u1 and u2, of U, as well as the input vector I(osc) orthogonalised with respect to these two principal components as basis for X. This basis retains 77% of the variance of X (measured by r2). Since now, similar to the low-rank case, we can write the projection of x(t) on I⊥(osc) as a function of θ, we can plot trajectories with coordinates (θ, u1Tx,u2Tx), and we obtain two stable cycles, linked in phase-space, as in Fig 2. B) Here we train full-rank networks without regularisation, and find dynamics lying on two non-linked cycles, similar to the rate-coding model in Fig 2. The basis is constructed similar to A), here explaining 87% of the variance. (TIF) [file pcbi.1011852.s002.tif]

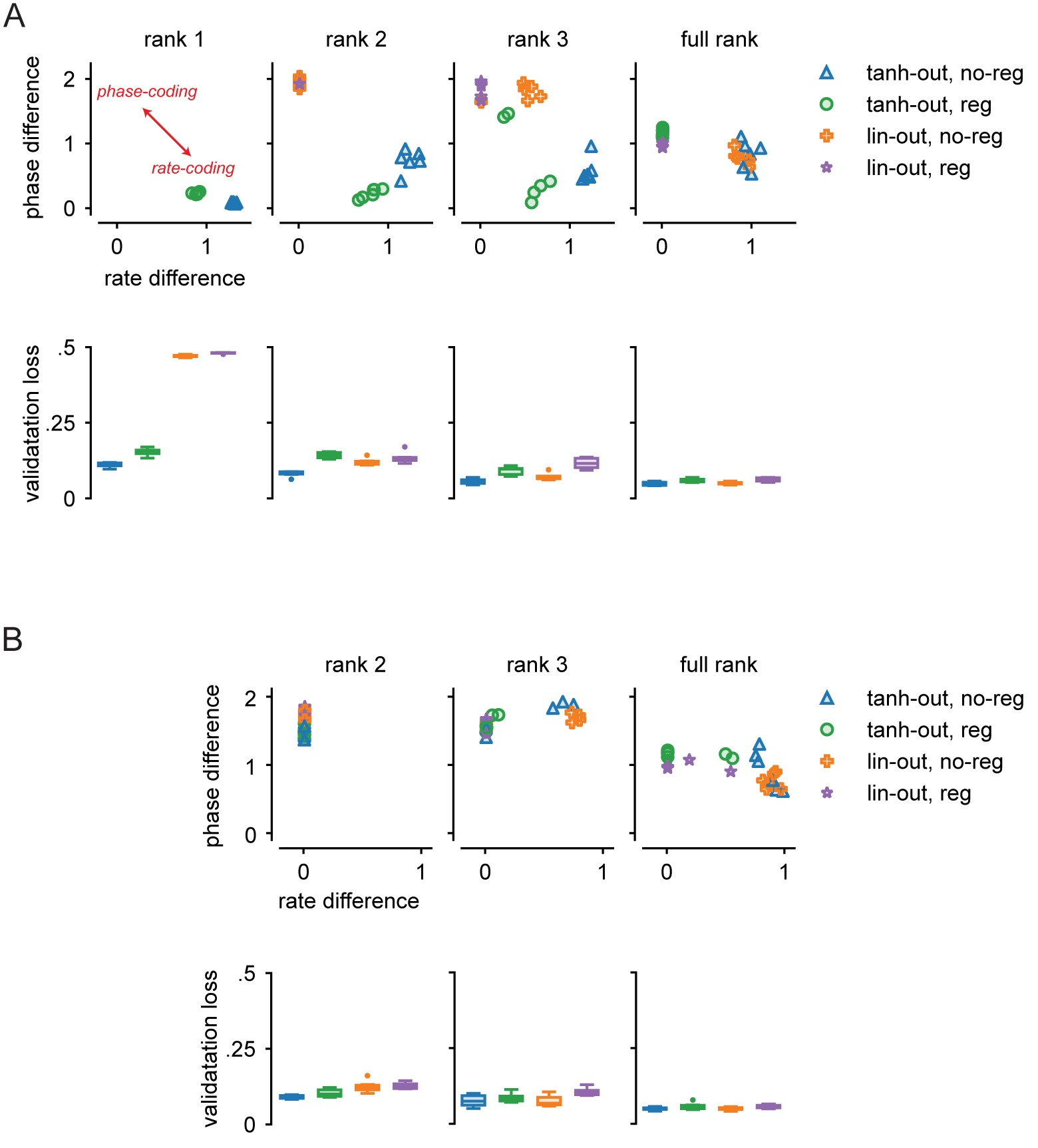

Supplement: S3 Fig — A) Here we analysed to what degree a model will learn a phase- versus a rate-coding solution, as a function of the training setup and initialisation. To quantify to what degree either solution is learned, we first computed for all units in a model the absolute difference between their (normalized) rate after stimulus a and their rate after stimuli b, as well as the absolute phase difference between trials with either stimulus. To get one measure of rate coding per model, we then calculated the mean over the absolute rate differences of all units, and similarly took the mean absolute phase differences as a measure of phase coding. We plot here in the first row these two measures for 6 models for each combination of the following conditions: ranks 1,2,3, full rank (columns); output during training is Wx (lin-out) or W tanh(x) (tanh-out); regularisation that penalises deviations from the mean (Eq 6) is used (reg) or not (no-reg). All of these models use the default initialisation used in the manuscript and were trained for 100 epochs. Additionally we plot the validation loss after training in the second row. We observe that for rank 1 only models with tanh-out learn the task, and that these models learn a purely rate-coding solution. This is in line with our theory, as we previously showed that the phase-coding models couples its autonomously generated oscillations. Autonomous oscillations can only be generated by models of rank 2 and higher. For rank 2 and higher we can see that regularised models with a linear readout during training will generally learn a phase-coding solution, whereas models with a non-linear readout and no regularisation will generally learn a rate-coding solution. B) We repeated the experiment, but now initialised each network to start out with oscillatory dynamics. We can do this by initialising the weight matrix with a pair of complex conjugate eigenvalues with real part larger than 1 [30, 42] (this is only possible for rank 2 and higher). We c [file pcbi.1011852.s003.tif]

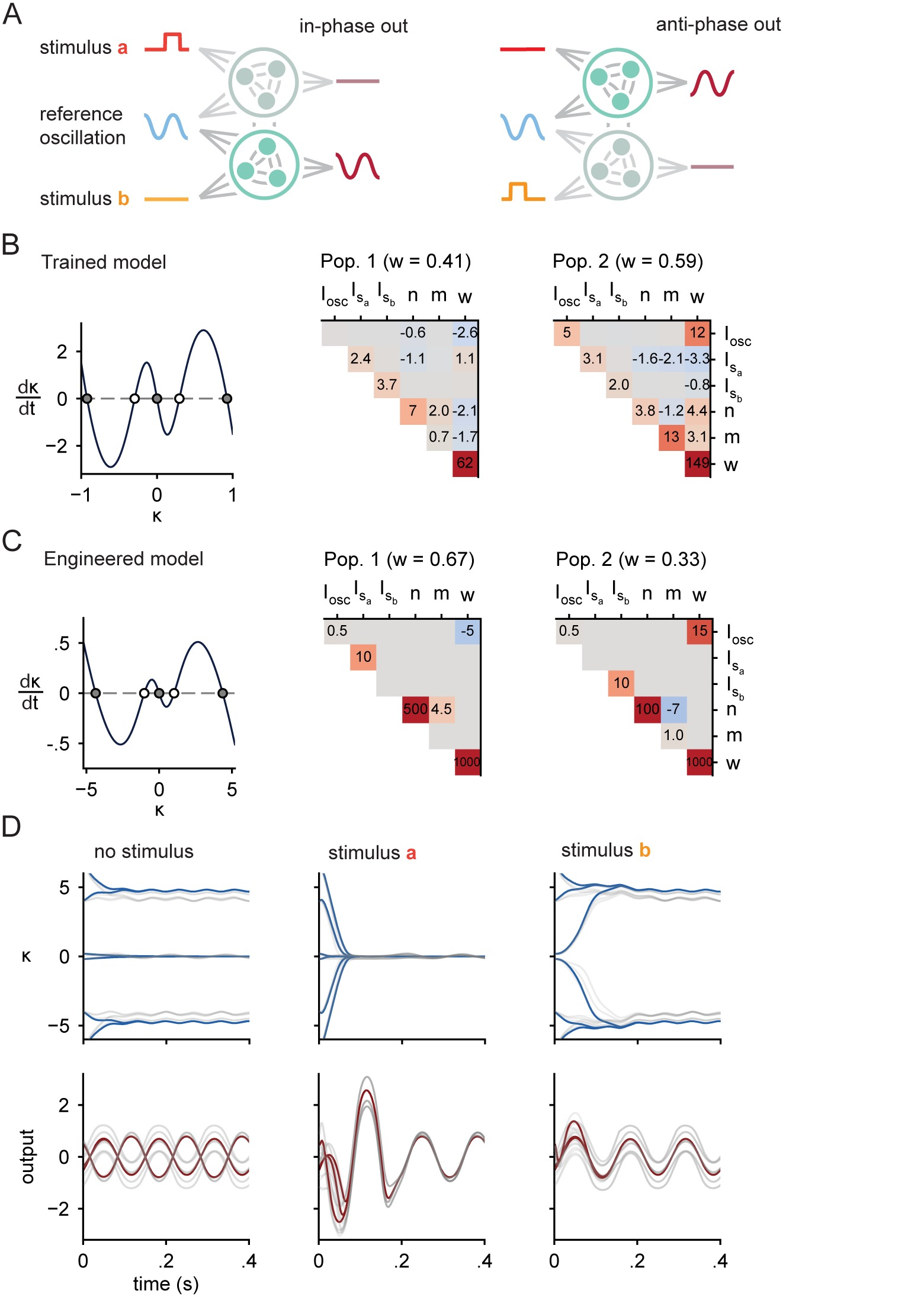

Supplement: S4 Fig — A) We here detail a simple rate-coding model that performs the working memory task. Such a model consists of two populations, one responsible for an oscillatory output in-phase with the reference oscillation, and one responsible for the anti-phase output oscillation. Depending on which stimulus was last seen, either population is dominating the readout. B) We reverse engineered a trained RNN with rank-1 connectivity. Due to the rank constraint, in the absence of any input, the trained model implements a one-dimensional dynamical system (with variable κ). We can plot the change in κ as a function of κ and observe the model learned to have three stable fixed points (left). We can fit a mixture of two Gaussians to the connectivity (I = input, n, m = left, right connectivity vector, w = output) which indicates that the model includes one population responsible for the fixed point at 0 (negative coupling between the connectivity vectors m, and n), while contributing little to the dynamics at the other fixed points (Due the large variance of m, the units of this population will have saturated rates when κ is away from 0). This population produces an in-phase oscillation, due to the positive covariance between input (I(osc)) and output weights (w). The other population is responsible for the two stable fixed points away from 0 (positive coupling between the connectivity vectors m, and n, small variance of m), and produces the anti-phase oscillation. Note that this model is almost identical to the one described in [30] Fig 4 (but with oscillations added on top). C) We can make the description from the previous panel more formal by engineering a rate-coding model. We initialised two covariance matrices (right), creating a two population model with similar dynamics as in the previous panel (left). D) We plot the latent dynamics (top row) and output (bottom row) given by the mean-field equation describing the network activity in the limit of infinite units (blue, red), as well [file pcbi.1011852.s004.tif]

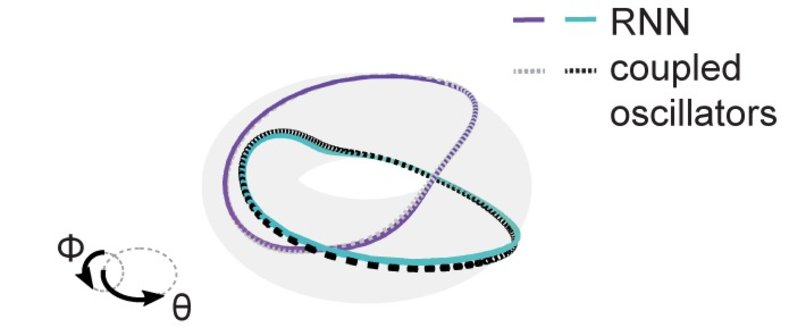

Supplement: S5 Fig — We simulated two coupled oscillators with the coupling function extracted from a trained network (Fig 3B). The two oscillators represent the RNN’s phase (ϕ) and the reference oscillation phase (θ). Starting simulations from various initial conditions demonstrates that the coupling function induces bistability, as all simulations converge to one of two stable cycles on the torus. Furthermore, the convergent trajectories of the coupled oscillators are a close match to those of the full RNN projected into the same space (ϕ, θ), indicating that the coupled oscillator description is appropriate for the RNN. (TIF) [file pcbi.1011852.s005.tif]

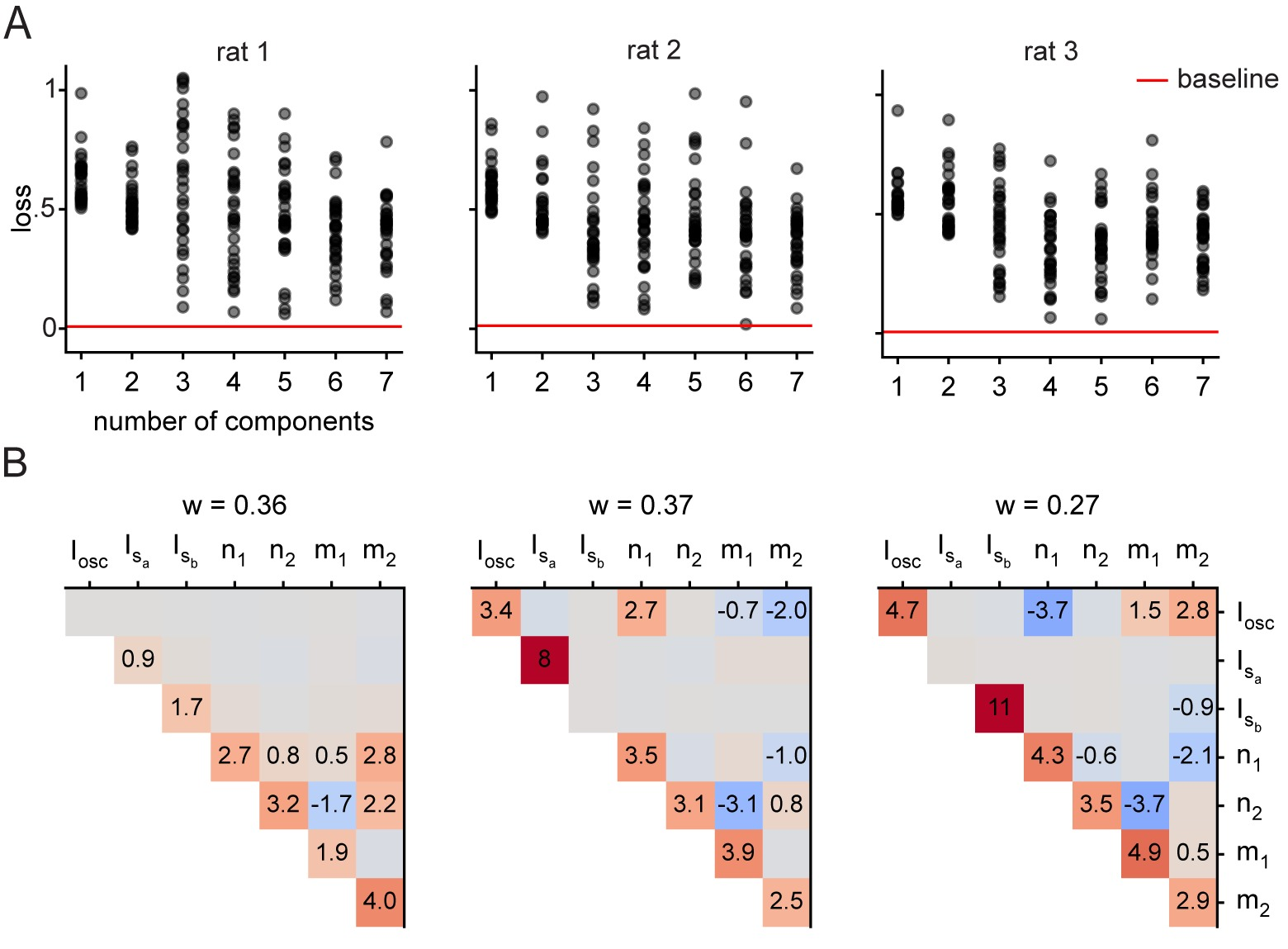

Supplement: S6 Fig — A) In order to study the connectivity of trained models, we fitted a mixture of Gaussians with 1 to 7 mixture components to the connectivity vectors of three different models [26], each trained with LFP data from a seperate rat. For this, we used variational inference with a Gaussian prior on the mean with precision 106 and mean 0. After each fit, we resampled the weights 30 times and computed the loss over a batch of 128 trials with a pure sine wave as reference oscillation. Altough for no amount of components we reliable were able to resample functioning models, from 3 components onwards a small fraction of sampled model have a comparable loss to the original trained model (red line). B) The covariance structure when fitting three components to weights of a trained network give us some hints as to what is needed to get a functioning model (top: covariances, bottom: pair plots). One component is unconnected (zero covariance) to the reference oscillation and has a skew-symmetric structure between the singular vectors. This structure generates oscillations [30, 42, 55]. The other two components, each connected to one stimulus and with opposite covariance between the input and singular vectors, together implement the coupling function. (TIF) [file pcbi.1011852.s006.tif]

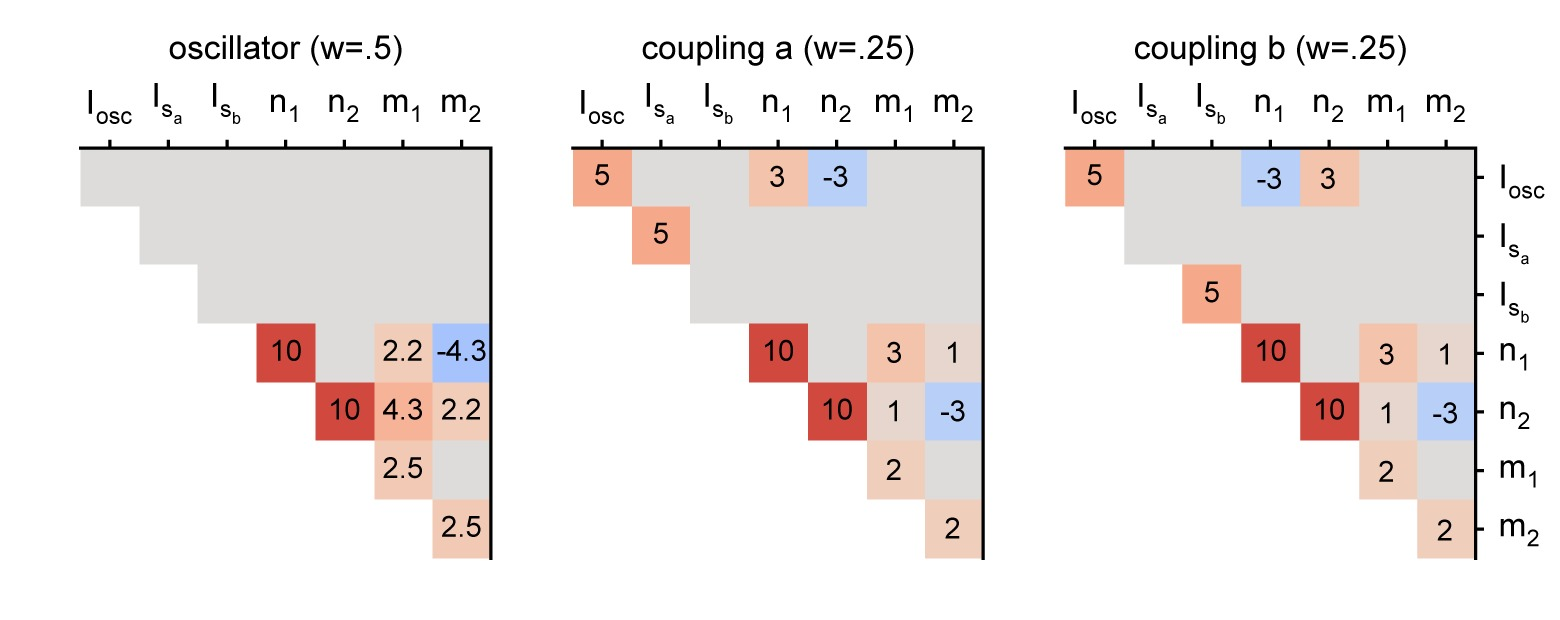

Supplement: S7 Fig — The designed covariance structure for the reduced model shown in Fig 4D and 4E, which leads to dynamics similar to the trained models. It also shares connectivity structure with trained models (S6B Fig), namely having one component unconnected to the reference oscillation that autonomously generates oscillations, and having the other two components, each connected to one stimulus, implement the coupling function. (TIF) [file pcbi.1011852.s007.tif]

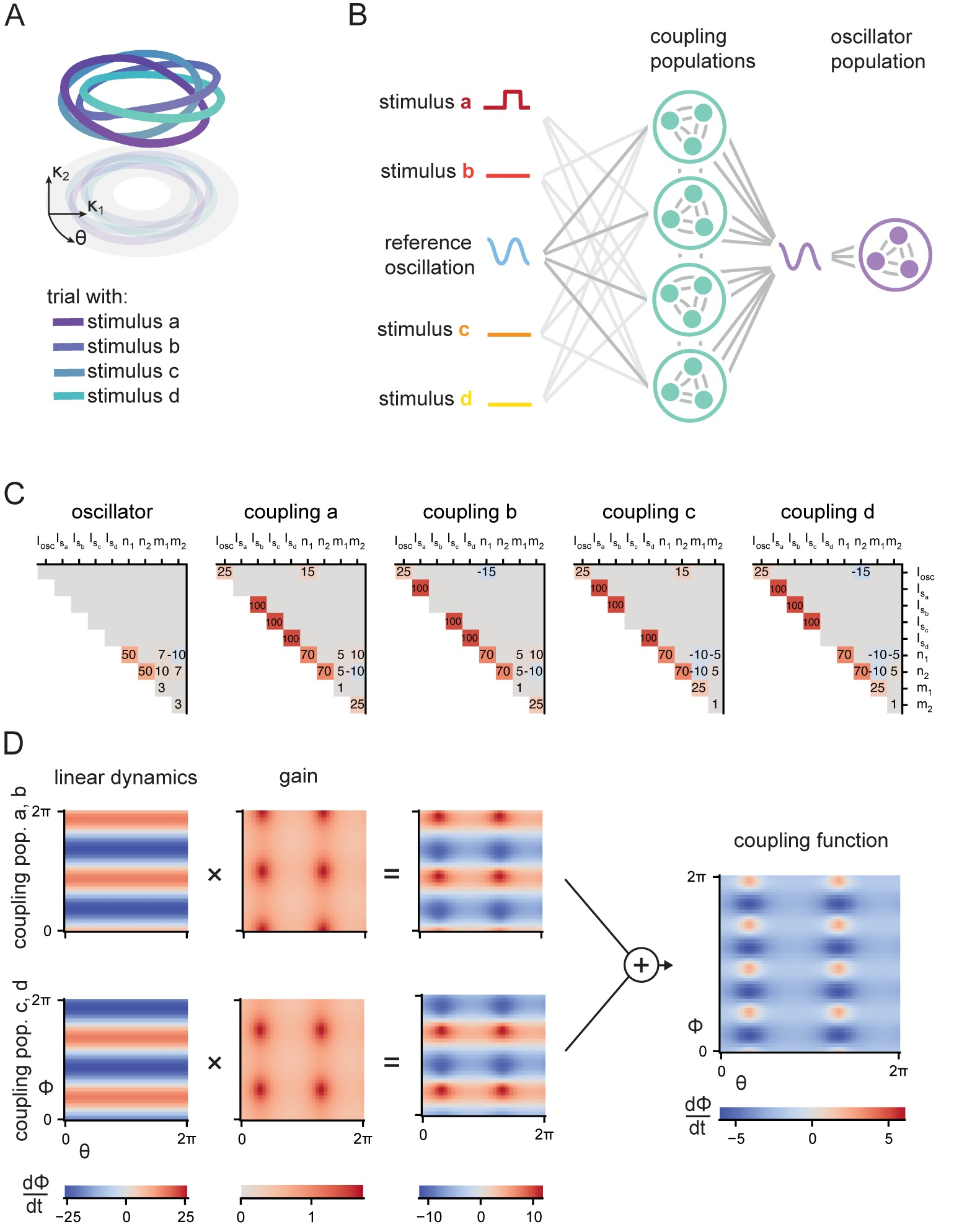

Supplement: S8 Fig — A) Our model can be straightforwardly extended to code for more than 2 stimuli. Here, we trained a network to maintain 1 of 4 stimuli at a given trial, by producing an output oscillation at −0.2π, −0.7π, −1.4π or −1.7π radians offset with respect to the reference LFP, for stimulus a, b, c or d, respectively. Again we find a stable limit cycle for each stimuli, which form linked cycles in phase-space. B) We now reverse engineered this model further, in particular we also found a reduced description of a model coding for 4 stimuli, in terms of 5 subpopulations. As before, 1 population generates oscillations, but we now have 4 coupling populations. Each stimulus inhibits all but one coupling population, so that the remaining coupling population guides the network dynamics to the limit cycle corresponding to the presented stimulus. C) The reduced model is specified by a mixture of 5 Gaussians, here we show the covariances that can be used to generate the connectivity of a model coding for 4 stimuli. D) Besides the connectivity with respect to the input, the recurrent dynamics (given by the overlaps, or covariances, between the connectivity vectors m’s and n’s) also had to be adjusted in order to allow memorising 4 stimuli instead of 2. We showed in our manuscript that rank 2 phase-coding models can described well by their coupling function, which gives the dynamics of the model as a function of the phase of the input oscillation (θ) and the phase of its internal oscillation (ϕ). The two limit cycles in the model described in the main text originated from the sin(2ϕ) and cos(2ϕ) terms in the coupling function. To code for 4 stimuli, we need higher frequency terms (e.g. cos(4ϕ)) in the coupling function. To understand how these originate from the recurrent connectivity, we decompose the coupling function. The coupling function generated by multiple populations is simply a summation over the coupling functions generated by the individual subpopulations. These in turn can b [file pcbi.1011852.s008.tif]

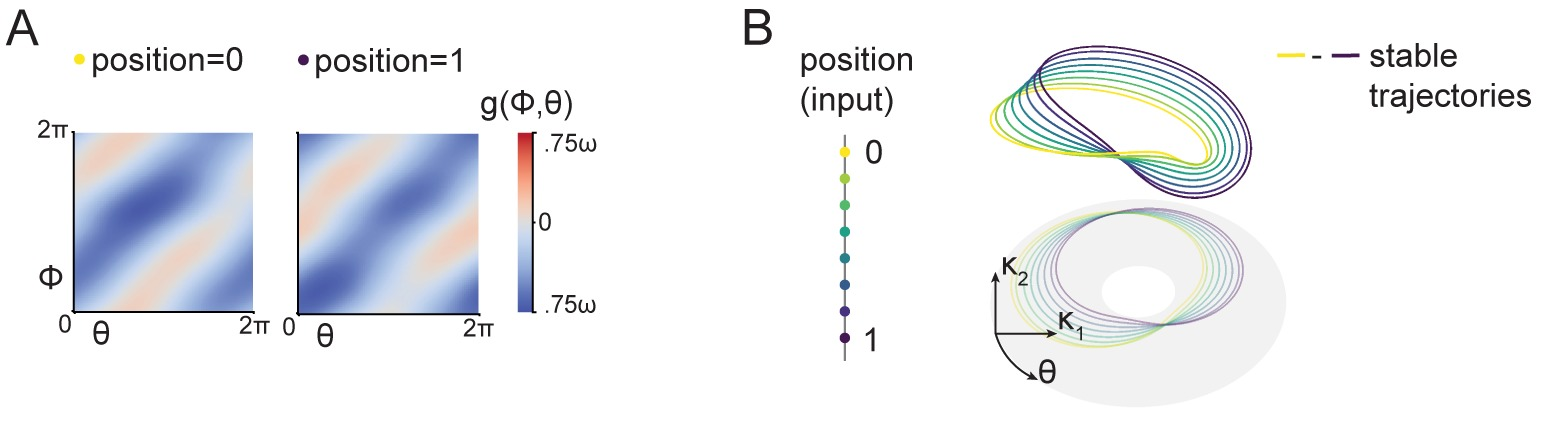

Supplement: S9 Fig — A) Phase precession in rat hippocampus entails a place cell changing its relative phase of firing as a result of rat moving through a place field [10]. We here show how to create a network that changes its relative phase of oscillation depending on a continuous valued stimulus input. We setup a network with connectivity drawn from a mixture of three Gaussians, again with two components implementing a coupling function and one component implementing an oscillator. The network receives a sinusoidal reference input with phase θ as sin(θ), cos(θ) through input vectors I(osca),I(oscb) respectively, as well as continuous valued stimulus input, representing place field position, s(t)∈[0, 1) as sin(s(t)12π),cos(s(t)12π) through input vectors I(sa),I(sb) respectively. The oscillator component has connectivity equal to S7 Fig, whereas for the coupling components (p2, p3) we define the covariance matrices as follows: For the off-diagonal elements, σn(1)I(oscb)(p2)=-σn(2)I(osca)(p2)=σn(2)I(osca)(p3)=σn(1)I(oscb)(p3), otherwise 0. For the diagonal elements, component two has zero variance for (is unconnected to) I(sa) and component three has zero variance for I(sb). This gives a coupling function of the form: g(θ,ϕ)=sin(θ-ϕ)ab+csin(π2s(t))2+cos(θ-ϕ)ab+ccos(π2s(t))2, for constants a, b, c that depend on the exact values of the variances and covariances of the mixture components. For large c and small b, the coupling function will change from a sin(θ − ϕ) to a cos(θ − ϕ) as s(t) changes from 0 to 1. We plotted here, the coupling function of a network (N = 2056 units) with connectivity drawn from mixture of Gaussians as described above, for s(t) = 0 and s(t) = 1. The coupling function translates between these two states as a consequence of position input s(t). B) Different trials on which s(t) is tonically presented at different values lead the the network locking to a unique phase difference with respect to the reference oscillation. In phase-space, this appears as the stable cycl [file pcbi.1011852.s009.tif]
