# Supplementary material for: Trained recurrent neural networks develop phase-locked limit cycles in a working memory task
Source: PLoS Comput Biol. 2024 Feb 5;20(2):e1011852. doi: 10.1371/journal.pcbi.1011852 (PMC10868787; doi:10.1371/journal.pcbi.1011852)
Supplement: Attachment — Submitted filename: ResponeToReviewers.pdf [file pcbi.1011852.s010.pdf]

Point by point response to reviews

Summary of changes

We thank the reviewers for their feedback. In particular, we thank both reviewers for their positive feedback on the presentation of our results, and pointing out that the results will be of broad interest to a computational audience. Based on the reviewers' suggestions, we added three supplementary Figures that: explain whether the models learn a rate- or phase-coding solution based on the initialisation and training setup (Rebuttal Fig. 1, Manuscript Fig. S3), explain the rate-coding model in more detail (Rebuttal Fig. 2, Manuscript Fig. S4), and detail a model coding for 4 stimuli (Rebuttal Fig. 3, Manuscript Fig. S8). In addition, we have updated the text in the manuscript based on the reviewer's feedback. Most importantly we clarified how our training setup relates to experiments, and what assumption we make by using local field potentials as input. We think this substantially increased the readability of our manuscript.

We here follow with a point by point response to each of their comments.

Response to reviewer #1

Minor questions and remarks

1. *The authors state there were two solutions found to solve the task – I wonder which one was more frequent? Most importantly, I wonder how the solutions were more generally affected by the applied connectivity constraints? Is there any lack in expressive power of the RNN through this approach? Or will certain solutions be favored over others?*

   The solution that the network ends up choosing arises from an interplay of the training setup and the initialisation. To gain some intuition on this, we ran additional analyses in which we trained 168 new models, and analysed for each model whether it ends up using a rate- or a phase code. The additional analysis show, among others, that a rank 1 network does not learn a phase-coding solution (in line with our hypothesis that phase-coding networks produce autonomous oscillations), whereas for rank 2 or higher both rate- and phase-coding solutions are found. The degree to which either solution is favored can to some degree be steered with regularisation and initialisation. We provide the details here in Rebuttal Fig. 1, and in Manuscript Fig. S3.

2. *Just to be sure I understood the rationale: the underlying (neurobiological) assumption of the way the task is composed, is that some part of the brain or neuronal subpopulation acts as an oscillatory reference signal (e.g., CA1), and that another population is coupled to this reference signal as well as external inputs, and by this can generate a second oscillatory signal (e.g., in-phase/out-of-phase, different limit cycles) used for phase-coding. The idea of the task is that the RNN is trained to produce this secondary signal? Or, in other words, what is the rationale behind making the RNN*

reproduce periodic behavior in response to the stimulus? Would appreciate to make this a bit clearer in the text, perhaps with a cognitive example.

You indeed got the rationale right, we thank you for clearly articulating the motivation behind our setup. Thanks to your suggestion we now explain more concretely how our model setup relates to existing experimental findings in the first section of the results, something that we think helped the readability of our manuscript.

To elaborate on the rationale here: Motivated by experiments in humans [1–3] and macaques [4], we aim to model a neural population maintaining a working memory by phase of firing relative to ongoing local field potential (LFP) oscillations. Our model relates to these experiments as follows:

- The transient pulse stimuli represent the working-memory item to be stored, e.g. a navigational goal [2], or sequence list position [1, 3].
- The oscillatory input represents the local field potential relative to which phase-coding was observed in experiments [1–5]. Oscillation in the LFP signal reflects the synchronised synaptic activity of large populations of neurons [6]. By using the LFP as input, we made the assumption that the neurons that we model (those that code for the working memory), have an overall negligble effect on the LFP. This can be the case if they are only a small subset of all neurons contributing to the LFP, or because the LFP reflects input coming from an upstream population e.g. from medial septum or CA1. This assumption is now explicitly stated in the manuscript. We also note that using theta oscillations as input to simulated neurons is in-line with previously proposed models [3, 7].
- The experimental observation is that firing of neurons preferentially happens in specific phases of the LFP oscillation. To capture this effect in a rate model, we demand that certain neurons in the population have an oscillatory firing rate with a desired phase to the LFP. We do so by training the output, which is a weighted sum of neuron activities.

3. *Can you quantify this statement: "Networks were able to successfully learn the task" (line 55). I guess in this application it is not necessary that all networks perform well, but the networks that do, should have a reasonably high "hit rate"?*

   In our study, we aimed to design the most simple task that allows us to study the mechanisms underlying phase-coding in RNNs. As a consequence, due to the simplicity of the task, all networks we used for the main Figures in the manuscript were successfully trained. For these models, we stopped training at 50 epochs, at which point the mean square error clearly converged (see Manuscript Fig. S1 for the loss curves). We now clarified this in the method section.

4. *Line 261: "10% served as validation set", so were the final analyzed models selected based on minimizing the test error?*

   As all models converged, we did not do any model selection. In Manuscript Fig. S1 we show the training and validation error are almost identical throughout training. This is expected, as the validation and training trials only differ in the portion of the local field potential that was used as reference signal for the network, and in the seed used for generating the randomised stimulus onsets and offsets. We updated the caption of S1, as well as the method section to clarify this.

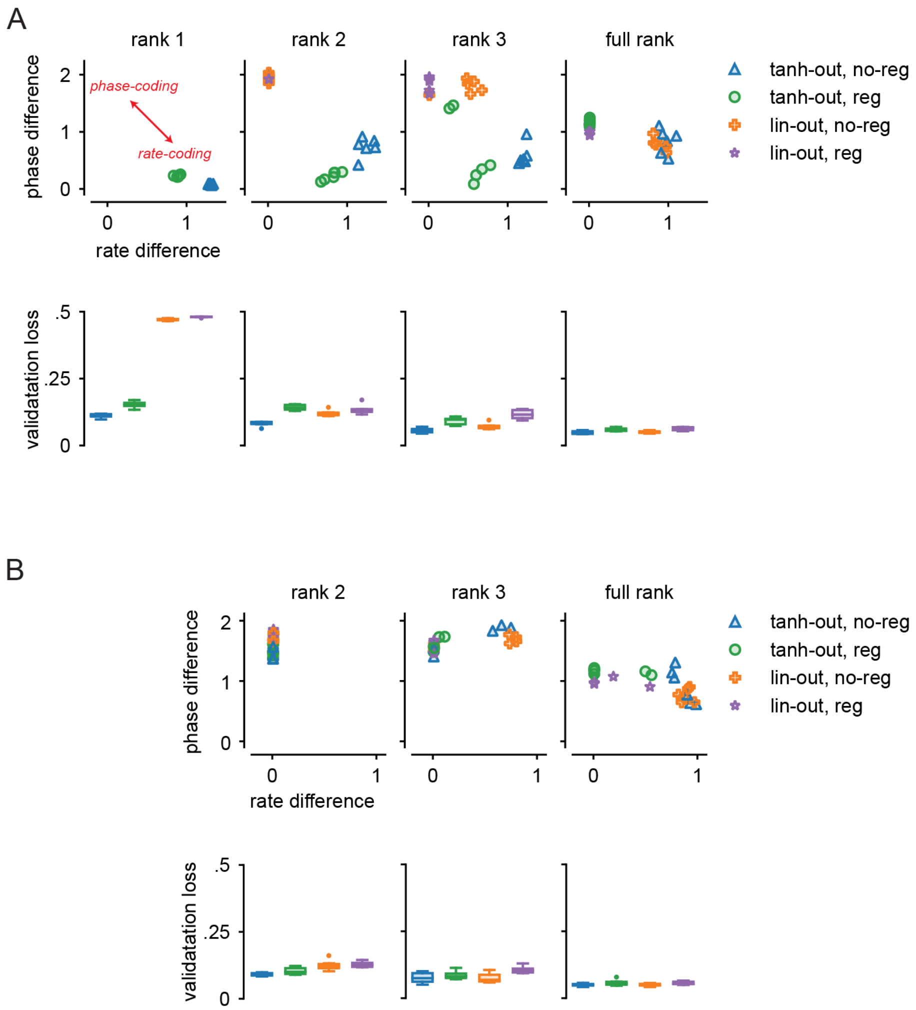

Fig 1. (*caption on next page*) **Influence of the training setup on the network solution.**

Fig 1. (*previous page*) **Influence of the training setup on the network solution.**

A) Here we analysed to what degree a model will learn a phase- versus a rate-coding solution, as a function of the training setup and initialisation. To quantify to what degree either solution is learned, we first computed for all units in a model the absolute difference between their (normalized) rate after stimulus a and their rate after stimuli b, as well as the absolute phase difference between trials with either stimulus. To get one measure of rate coding per model, we then calculated the mean over the absolute rate differences of all units, and similarly took the mean absolute phase differences as a measure of phase coding. We plot here in the first row these two measures for 6 models for each combination of the following conditions: ranks 1,2,3, full rank (columns); output during training is $\mathbf{W}\mathbf{x}$ (*lin-out*) or $\mathbf{W}\tanh(\mathbf{x})$ (*tanh-out*); regularisation that penalises deviations from the mean (Manuscript Eq. 6) is used (*reg*) or not (*no-reg*). All of these models use the default initialisation used in the manuscript and were trained for 100 epochs. Additionally we plot the validation loss after training in the second row. We observe that for rank 1 only models without regularisation learn the task, and that these models learn a purely rate-coding solution. This is in line with our theory, as we previously showed that the phase-coding models couples its autonomously generated oscillations. Autonomous oscillations can only be generated by models of rank 2 and higher. For rank 2 and higher we can see that regularised models with a linear readout during training will generally learn a phase-coding solution, whereas models with a non-linear readout and no regularisation will generally learn a rate-coding solution.

B) We repeated the experiment, but now initialised each network to start out with oscillatory dynamics. We can do this by initialising the weight matrix with a pair of complex conjugate eigenvalues with real part larger than 1 [8, 9] (this is only possible for rank 2 and higher). We can observe that models with oscillatory initialisation are generally more likely to learn a phase-coding solution; in particular all rank 2 models learn a purely phase-coding solution.

Response to reviewer #2

Major comments

1. *line 69: what does "depending on the training setup" mean? are these different solutions just due to the network's random initialization? (coming back to this later:) Fig S3 seems to explain it. am i correct in understanding that the network's solution is totally determined by whether there is a nonlinearity in the objective? as the results are presently presented in the main text, it's not clear that the authors understand why the two solutions occur, so I think it would strengthen their presentation to go into this more in the main text.*

   The solution the network ends up going for indeed depends on whether the output of the network includes the non-linearity, but also on the initialisation, rank constraint and regularisation applied during training. To get more insight in this question, we now trained 168 new models, and analysed whether they end up using a rate-coding or a phase-code. (Rebuttal Fig. 1, Manuscript Fig. S3). Indeed, rank 2 models without non-linearity in the readout will generally learn a phase-coding solution, whereas rank 2 models with a non-linear readout will generally learn a rate-coding solution. However, as we now also show, when we initialise rank 2 models with oscillations (i.e. with a recurrent weight matrix with a pair of complex conjugate, instead of two real eigenvalues), all rank 2 learn a phase-coding solution irrespective of how the readout is formulated. The situation for rank 3 and higher is again slightly different (e.g. whether a model learns a phase or rate-coding solution for full rank networks is independent of the readout, but depends on the regularisation). Given that the situation is so complicated, and not the focus of our paper, we decided to keep this analysis to the supplementary. We do now point the reader to it, when we introduce the fact that there are two solutions in the main text.

2. *The authors mention that the second solution resembles "previous work on fixed point dynamics in RNNs." i didn't really follow what this means. in general, this felt like a pretty brief dismissal of one of the two identified network solutions.*

   Based on your suggestion, we performed new analyses to reverse engineer a model using the rate-coding solution (details in: Rebuttal Fig. 2, Manuscript Fig. S4). This model works by having two subpopulations, each active at different locations in phase space. Depending on which stimulus was last seen, either subpopulation dominates the readout. One subpopulation directly transmit the input oscillation, creating an in-phase oscillation, whereas the other population flip the sign of the input oscillation, creating an anti-phase oscillation.

   To make the comparison with previous work more concrete, we obtained a reduced (meanfield) model (Rebuttal Fig. 2C), which turns out to be essentially the same model as described in [9] (2 populations, one creating

a stable fixed point at the origin, and one creating stable fixed points away from the origin), with the input oscillation superimposed.

Minor comments

1. *could the authors add to the results text a brief rationale for why the network is provided with an oscillatory reference input (e.g., near equation 1)?*

2. The oscillatory input represents the oscillatory local field potential relative to which phase-coding was observed in experiments [1–5]. By using these oscillations as input, we made the assumption that the neurons that we model (those that code for the working memory), have an overall negligable effect on the ongoing oscillations. This can be the case if they are only a small subset of all neurons contributing to the population oscillation, or because these oscillations reflects input coming an upstream population. This assumption is now explicitly named in the manuscript. We also note that using theta oscillations as input to simulated neurons is in-line with previously proposed models [3, 7].

3. *line 50: here the paper introduces the use of LFPs. could the authors provide some context for why this is the right thing to include in the network? also, does the inclusion of LFPs in u(t) mean we should think of x(t) as also being an LFP signal? or should x(t) be thought of as firing rates? (i guess my general point is that it could be helpful to have some high-level/systems-level explanation of how the authors are imagining LFPs and firing rates to interact.)*

   We use local field potentials, in the first place, as in the experiments we based our model on, coding was shown by phase of firing relative to local field potential oscillations [1–5]. Concretely, in our model, we make the assumption that the local field potentials, u(t), influence the neurons that are coding for the stimulus (which are described by currents (x(t), and firing rates tanh(x(t)), but, as motivated above, that these neurons themselves have have a negligible effect on the local field potential - thus making it valid to use LFPs as input to the model.

4. *Fig 2A and Fig 2D took me a long time to parse. It was confusing to me that the caption for Fig 2A referred to the "top row" but not the "bottom row", because I assumed "top row" meant the top row _of Fig 2A_, meaning the top two units. it would also help to add a text title to Fig 2A that says "Solution 1" and another one to Fig 2D that says "Solution 2." And then label each subpanel as Unit 1, Unit 2, etc.*

   We apologize for the confusion caused, and have now updated the Figure based on your suggestion. We additionally replaced "top row", with "panels A-C" and "bottom row" with "panels D-F" to avoid any future confusion.

5. *after seeing Fig 2 (which I found really clear and interesting, once I understood what the subpanels meant), i found myself wondering how I can interpret these two solutions as valid solutions given the network's objective. currently, the training objective is only presented in methods. it could be helpful to have some explanation of the objective in results, and then help the reader understand why the two network solutions "make sense" given this objective. for example, is it possible to explain why the unit activity in Fig 2D is a reasonable solution to this task?*

   In the model shown in Fig 2D, a different subset of units has activity far away from 0 after either stimulus. These units saturate their (i.e. are at the flat part of their) tanh non-linearity, and as a consequence have little effect on the phase of the output oscillation. As a different subset of units saturates after after either stimulus, different units determine output oscillation. These units either directly transmit the input oscillation, creating an in-phase oscillation, or flip its sign to create an anti-phase oscillation. Based on your suggestion, we updated the main results text to clarify this point.

6. *the explanation of poincare maps and the cross-section Q went over my head. the function (?) P in the equation after line 85 is also not defined. also, the vector K_c is in S, but I don't know what S is, nor does I know what an intersection is. I'm not sure if me understanding all of these things is essential for me to get the point, so you could potentially move this (along with more details) to methods. but either way a little more hand-holding would help a lot.*

   We apologize for not clearly describing the Poincaré maps. Indeed S was not defined anywhere, we now omitted this. While indeed understanding all the details is not necessary for getting the main point (a method to study the stability of limit cycles), we do want to keep some description of Poincaré maps in the main text as we believe this is a method is not widely used by, but that can be useful for other researchers studying models of oscillatory dynamics. We now updated the description in the manuscript and link to the full equation for the Poincaré map $\mathbf{P}$ in the methods.

7. *Fig 3 as a whole is an excellent graphical depiction of what is going on, and the text for this part is, on the whole, very clear. loved it!*

   We thank you for your positive feedback!

8. *do the authors know what the importance of the network having rank 2 dynamics is? i am curious why the authors chose that particular rank, and also if this "matters" in the sense of the network solutions. e.g., does solution 1 vs 2 dominate if the dynamics had rank 1, or rank 3, etc?*

   In Rebuttal Fig.1 and in Manuscript Fig S3. we now perform a more in-depth investigation of the solution as a function of rank of the model. We can see that in rank 1 models only the rate coding solution exists, which is

perfectly inline with our theory — we previously showed that the phase-coding model produces autonomous oscillations (which it couples to the reference), generating oscillations is only possible from rank 2 onwards. The importance of rank 2, is that it is the minimal rank for which the phase-coding solution exists, and thus in some sense the simplest possible model we can analyse. We also show that both solutions exist in full-rank networks (Manuscript Fig. S2).

9. *the model shown in Fig 4D had me wondering how easily this would scale to multiple stimuli. e.g., from Fig 4D it seems like you'd need a new coupling population (or maybe even more) for a third stimulus–is this true? i see that Fig S7 shows this is possible in principle. but could the authors explain how their model needs to change to accommodate more stimuli, e.g., does the model in Fig 4D need to be changed?*

   To answer you question, we now obtained a new model coding for 4 stimuli with weights drawn from a mixture of Gaussians (Rebuttal Fig.3, Manuscript Fig. S8). You are indeed correct that this model needs additional coupling populations. While we show that 2 coupling populations are in principle enough to get recurrent dynamics with 4 limit cycles, in order to be able to properly respond to the 4 different input stimuli, this models ends up having 4 coupling populations.

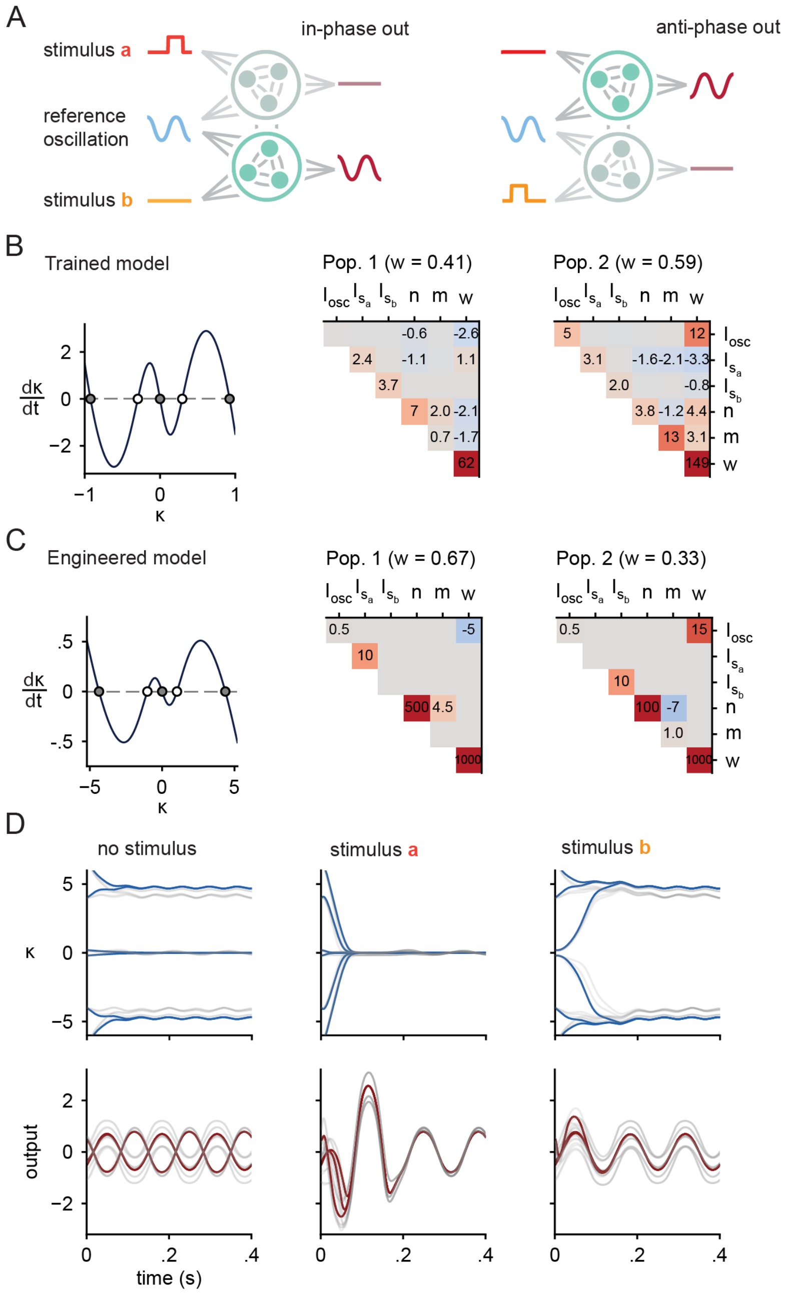

Fig 2. (*caption on next page*) **Rate-coding solution in detail.**

Fig 2. (*previous page*) **Rate-coding solution in detail.**

A) We here detail a simple rate-coding model that performs the working memory task. Such a model consists of two populations, one responsible for an oscillatory output in-phase with the reference oscillation, and one responsible for the anti-phase output oscillation. Depending on which stimulus was last seen, either population is dominating the readout.

B) We reverse engineered a trained RNN with rank-1 connectivity. Due to the rank constraint, in the absence of any input, the trained model implements a one-dimensional dynamical system (with variable κ). We can plot the change in κ as a function of κ and observe the model learned to have three stable fixed points (left). We can fit a mixture of two Gaussians to the connectivity ($\mathbf{I}$ = input, $\mathbf{n}, \mathbf{m}$ = left, right connectivity vector, $\mathbf{w}$ = output) which indicates that the model includes one population responsible for the fixed point at 0 (negative coupling between the connectivity vectors $\mathbf{m}$, and $\mathbf{n}$), while contributing little to the dynamics at the other fixed points (Due the large variance of $\mathbf{m}$, the units of this population will have saturated rates when κ is away from 0). This population produces an in-phase oscillation, due to the positive covariance between input ($\mathbf{I}^{(osc)}$) and output weights ($\mathbf{w}$). The other population is responsible for the two stable fixed points away from 0 (positive coupling between the connectivity vectors $\mathbf{m}$, and $\mathbf{n}$, small variance of $\mathbf{m}$), and produces the anti-phase oscillation. Note that this model is almost identical to the one described in [9] Figure 4 (but with oscillations added on top).

C) We can make the description from the previous panel more formal by engineering a rate-coding model. We initialised two covariance matrices (right), creating a two population model with similar dynamics as in the previous panel (left).

D) We plot the latent dynamics (top row) and output (bottom row) given by the mean-field equation describing the network activity in the limit of infinite units (blue, red), as well as finite size simulations (grey). For the finite size simulations, we used models (N=4096) with weights sampled from the mixture of Gaussians. We can see that in the absence of input, depending on the initial state the model will go to either the fixed point at 0, corresponding to an in-phase oscillation, or one of the two fixed points away from 0, corresponding to an anti-phase oscillation, respectively. For the second and third column, we provided the network with stimulus a, and b respectively, for the first .15s of the trial. Stimuli can successfully steer the network to the right fixed point and output oscillation phase, irrespective of initial condition.

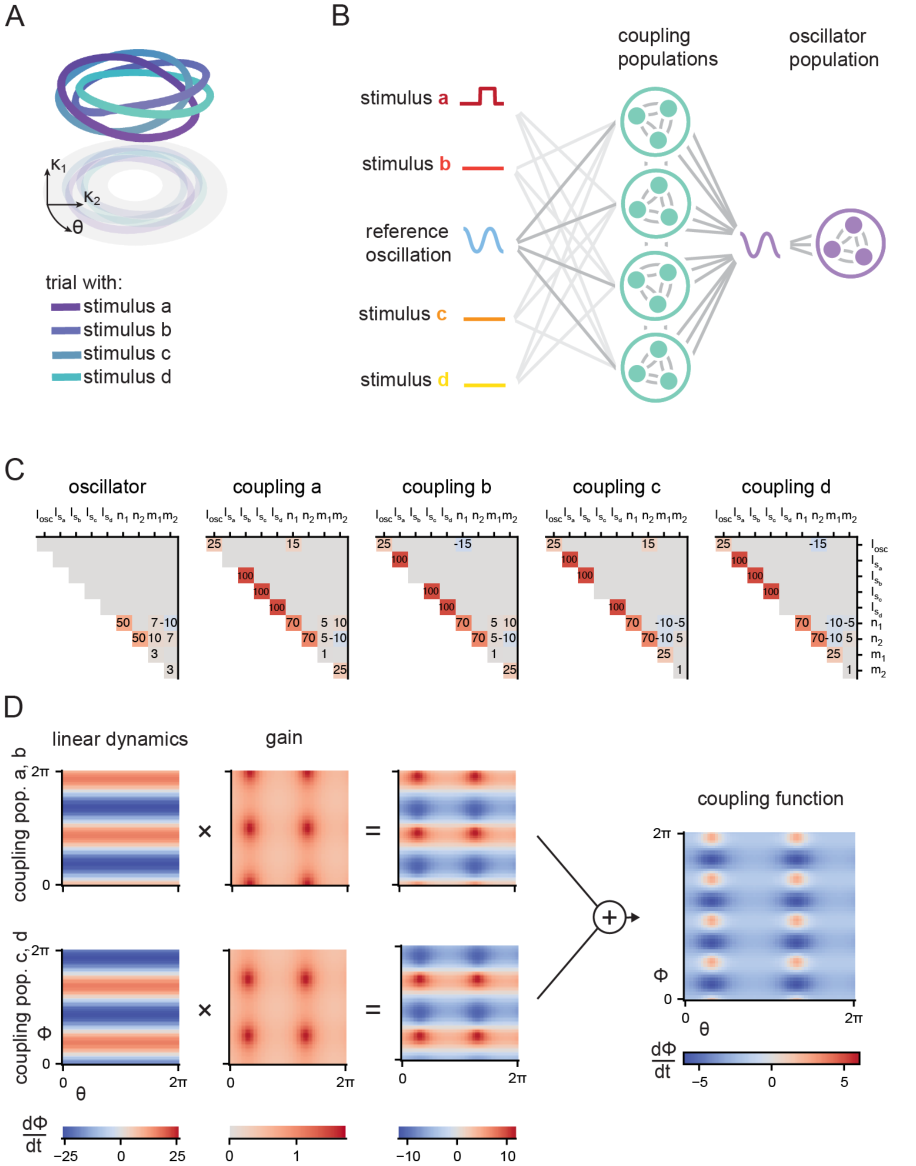

Fig 3. (*caption on next page*) **Geometry is preserved in a model coding for 4 stimuli, but more coupling populations are required.**

Fig 3. (*previous page*) **Geometry is preserved in a model coding for 4 stimuli, but more coupling populations are required.**

A) Our model can be straightforwardly extended to code for more than 2 stimuli. Here, we trained a network to maintain 1 of 4 stimuli at a given trial, by producing an output oscillation at $-0.2\pi, -0.7\pi, -1.4\pi$ or -1.7π radians offset with respect to the reference LFP, for stimulus a, b, c or d, respectively. Again we find a stable limit cycle for each stimuli, which form linked cycles in phase-space.

B) We now reverse engineered this model further, in particular we also found a reduced description of a model coding for 4 stimuli, in terms of 5 subpopulations. As before, 1 population generates oscillations, but we now have 4 coupling populations. Each stimulus inhibits all but one coupling population, so that the remaining coupling population guides the network dynamics to the limit cycle corresponding to the presented stimulus.

C) The reduced model is specified by a mixture of 5 Gaussians, here we show the covariances that can be used to generate the connectivity of a model coding for 4 stimuli.

D) Besides the connectivity with respect to the input, the recurrent dynamics (given by the overlaps, or covariances, between the connectivity vectors $\mathbf{m}$'s and $\mathbf{n}$'s) also had to be adjusted in order to allow memorising 4 stimuli instead of 2. We showed in our manuscript that rank 2 phase-coding models can described well by their coupling function, which gives the dynamics of the model as a function of the phase of the input oscillation (θ) and the phase of its internal oscillation (ϕ). The two limit cycles in the model described in the main text originated from the $\sin(2\phi)$ and $\cos(2\phi)$ terms in the coupling function. To code for 4 stimuli, we need higher frequency terms (e.g. $\cos(4\phi)$) in the coupling function. To understand how these originate from the recurrent connectivity, we decompose the coupling function. The coupling function generated by multiple populations is simply a summation over the coupling functions generated by the individual subpopulations. These in turn can be decomposed as the product of a linear part stemming from the covariances between the connectivity $\mathbf{m}$'s and $\mathbf{n}$'s of the subpopulations, and a non-linear part (the gain) that relates to how saturated neurons in this populations rates are (a neuron is saturated when its activity is at the flat part of the tanh non-linearity). Here we show that two populations that saturate at opposite phases of ϕ and θ are enough to create a coupling function containing higher frequency terms, leading to dynamics with four limit cycles.

References

[1] Liebe S, Niediek J, Pals M, Reber TP, Faber J, Bostroem J, et al.. Phase of firing does not reflect temporal order in sequence memory of humans and recurrent neural networks; 2022. bioRxiv:2022.09.25.509370 [Preprint]. Available from: `https://www.biorxiv.org/content/10.1101/2022.09.25.509370v1`.

[2] Watrous AJ, Miller J, Qasim SE, Fried I, Jacobs J. Phase-tuned neuronal firing encodes human contextual representations for navigational goals. eLife. 2018;7:e32554. doi:10.7554/eLife.32554.

[3] Kamiński J, Brzezicka A, Mamelak AN, Rutishauser U. Combined Phase-Rate Coding by Persistently Active Neurons as a Mechanism for Maintaining Multiple Items in Working Memory in Humans. Neuron. 2020;106(2):256–264.e3. doi:10.1016/j.neuron.2020.01.032.

[4] Siegel M, Warden MR, Miller EK. Phase-dependent neuronal coding of objects in short-term memory. Proceedings of the National Academy of Sciences. 2009;106(50):21341–21346. doi:10.1073/pnas.0908193106.

[5] Turesson H, Logothetis N, Hoffman K. Category-selective phase coding in the superior temporal sulcus. Proceedings of the National Academy of Sciences. 2012;109(47):19438–19443. doi:10.1073/pnas.1217012109.

[6] Buzsáki G, Anastassiou CA, Koch C. The origin of extracellular fields and currents —EEG, ECoG, LFP and spikes. Nature Reviews Neuroscience. 2012;13(6):407–420. doi:10.1038/nrn3241.

[7] Lisman JE, Idiart MAP. Storage of 7 ± 2 Short-Term Memories in Oscillatory Subcycles. Science. 1995;267(5203):1512–1515. doi:10.1126/science.7878473.

[8] Mastrogiuseppe F, Ostojic S. Linking Connectivity, Dynamics, and Computations in Low-Rank Recurrent Neural Networks. Neuron. 2018;99(3):609–623.e29. doi:10.1016/j.neuron.2018.07.003.

[9] Beiran M, Dubreuil A, Valente A, Mastrogiuseppe F, Ostojic S. Shaping Dynamics With Multiple Populations in Low-Rank Recurrent Networks. Neural Computation. 2021;33(6):1572–1615. doi:10.1162/neco_a_01381.
